# On the possibility of death of new genes – evidence from the deletion of de novo microRNAs

**DOI:** 10.1186/s12864-018-4755-1

**Published:** 2018-05-23

**Authors:** Guang-An Lu, Yixin Zhao, Zhongqi Liufu, Chung-I Wu

**Affiliations:** 1 State Key Laboratory of Biocontrol, School of Life Sciences, Sun Yat-sen University, Guangzhou, 510275 Guangdong China; 2 Department of Ecology and Evolution, University of Chicago, Chicago, Illinois 60637 USA

**Keywords:** de novo gene, New gene, microRNA, Gene death, *Drosophila*

## Abstract

**Background:**

New genes are constantly formed, sometimes from non-genic sequences, creating what is referred to as de novo genes. Since the total number of genes remains relatively steady, gene deaths likely balance out new births. In metazoan genomes, microRNAs (miRs) genes, small and non-coding, account for the bulk of functional de novo genes and are particularly suited to the investigation of gene death.

**Results:**

In this study, we discover a *Drosophila*-specific de novo miRNA (*mir-977*) that may be facing impending death. Strikingly, after this testis-specific gene is deleted from *D. melanogaster*, most components of male fitness increase, rather than decrease as had been expected. These components include male viability, fertility and males’ ability to repress female re-mating. Given that *mir-977* has a negative fitness effect in *D. melanogaster*, this de novo gene with an adaptive history for over 60 Myrs may be facing elimination. In some other species where *mir-977* is not found, gene death may have already happened.

**Conclusion:**

The surprising result suggests that de novo genes, constantly rising and falling during evolution, may often be transiently adaptive and then purged from the genome.

**Electronic supplementary material:**

The online version of this article (10.1186/s12864-018-4755-1) contains supplementary material, which is available to authorized users.

## Background

Perhaps the most surprising insight to emerge from evolutionary genomics is that while genome sizes fluctuate over several orders of magnitude, gene content remains stable over long evolutionary periods. Given this observation, it is tempting to assume that new genes seldom arise in genomes. However, numerous studies, beginning with [1] and others [2–5], suggest that gene duplication at least is a prominent source of new genes. While emergence of new genetic material from components of existing genes is now widely accepted, the idea that protein-coding sequences can spring de novo from non-coding regions is more controversial. While some examples have recently been obtained [6–9], it is more likely that non-coding genes can originate this way since they would not have to produce functional and folding proteins. Indeed, systematic studies of non-coding RNA evolution found frequent de novo generation of such genes [10–16]. Given that despite this frequent generation of novel material gene content remains stable over time, one has to conclude that genes are equally frequently lost [6, 17].

A common form of gene death is akin to “Dead On Arrival” transposons [18, 19], i.e. genes that never truly become functional and are expressed as transcription noise [20]. Although some examples of the elimination of established new protein-coding genes are available [21–26], these observations were made after the loss of transcription or function has been completed. In contrast, we are interested in studying the processes that lead to de novo gene death, an aspect of genome ecology that has been largely ignored.

To study the processes that lead to the death of established new genes, it would be very informative to capture genes as they are being eliminated from the genome. The difficulty is that it is hard to predict from sequence data alone when a gene is on its way out. In a previous survey [11] we found a cluster of eight miRNAs that arose de novo in *Drosophila*, evolved conservatively for millions of years, but whose evolution sped up in more recent lineages. Although it is tempting to think that these miRNA loci are undergoing gene death, it is also possible that they are adaptively evolving after a shift in their function. To test these possibilities, we deleted one of these miRNAs from the genome and tested the effect of the knockout on a number of fitness components. We found no evidence of a novel function, but abundant reasons to believe that the deletion genotype is more fit that the functional copy, suggesting that this gene is indeed undergoing elimination. Our results suggest that gene death is an important component of genome evolution that can be studied by combining sequence and functional approaches.

## Results

### Fast sequence and expression evolution of *mir-977*

**Fig. 1:**
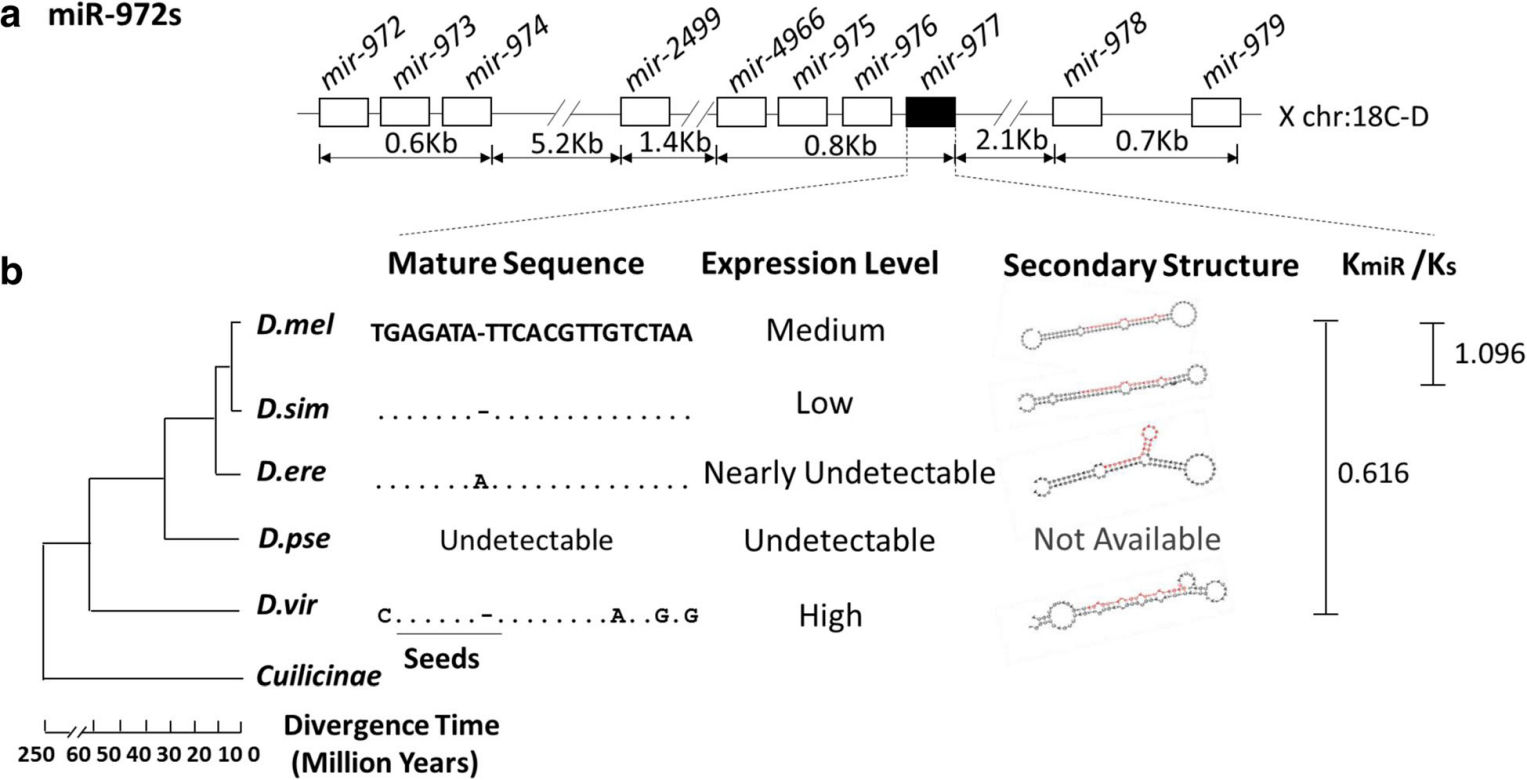
*mir-977* evolution. **a** *mir-977* genomic location. **b** *mir-977* sequence and expression level evolution. *mir-977* have fast sequence evolution and strong expression divergence among species. Species abbreviations: *D. mel*: *D. melanogaster; D. sim*: *D. simulans; D.ere: D. erecta D. pse*: *D. pseudoobscura; D. vir*: *D. virilis.* Expression level categories are: high, RPM > 5000; medium, RPM > 500; low, RPM > 50; nearly undetectable: RPM < 50

The inference of the origination of *mir-977* is based on the phylogenetic history of the entire cluster shown in Fig. 1. With three mosquito (*Culicinae*) genomes as outgroup (see [11]) together with all the in-group *Drosophila* genomes, every member of the miR- 972 cluster can be placed on the phylogenetic tree in the context of the 10 miRNA genes of the cluster. This is a cluster that has a “track record” of generating de novo miRNAs continually [10, 11] . We now stated the de novo origin of *mir-977* with greater circumspection in the context of other younger de novo miRNAs.

Focusing on the *mir-977* locus from the miR-972 cluster, we examined its nucleotide substitution rates among *Drosophila* lineages (Fig. 1a). This X-linked gene originated de novo prior to the origin of the *Drosophila* clade (more than 60 Myrs ago). The seed sequence in its mature product is almost identical between the *melanogaster* and *virilis* groups. However, younger *Drosophila* lineages show much faster evolution. Indeed, while the conservation statistic is 0.616 between *D. virilis* and *D. melanogaster*, it is 1.096 between *D. simulans* and *D. melanogaster* [11] .The 1.8 fold change is significant, (*P* < 0.05, Fisher’s exact test)*.* Furthermore, this gene was altogether lost in the *D. pseudoobscura* lineage [11].

In addition to its fast sequence evolution, expression levels of *mir-977* have also diverged among species. Using public small miRNA-seq data (see Methods and Additional file 1: Table S1), we found that *mir-977* exhibits high and medium expression in *D. melanogaster* and *D. virilis* but rather low and nearly undetectable expression in *D. simulans* and *D. erecta*. The nearly undetectable expression in *D. erecta* might be due to multiple hairpins in its secondary structure, which would lead to abnormal miRNA processing. Furthermore, *mir-977* has higher expression variance in the testes of multiple *D. melanogaster* lines than the conserved miRNAs that have important developmental functions (miR-184, bantam etc.; see Additional file 1: Table S2). Given its disappearance in the *D. pseudoobscura* and *D. erecta* lineages, fast evolution of *mir-977* sequence and expression levels in *D. melanogaster* indicate its impending elimination from that genome as well.

### *mir-977* gene deletion

To further investigate the possible fate of *mir-977* in *D. melanogaster*, we wanted to assay its effect on fitness. A deletion of this miRNA at its locus is necessary to perform robust experiments of this sort. While a large collection of targeted knockouts of *D. melanogaster* miRNAs does exist [27], the deletion that eliminates *mir-977* in that set also disrupts other loci. Therefore, we set out to generate a specific knockout of *mir-977* in *D. melanogaster*.

**Fig. 2:**
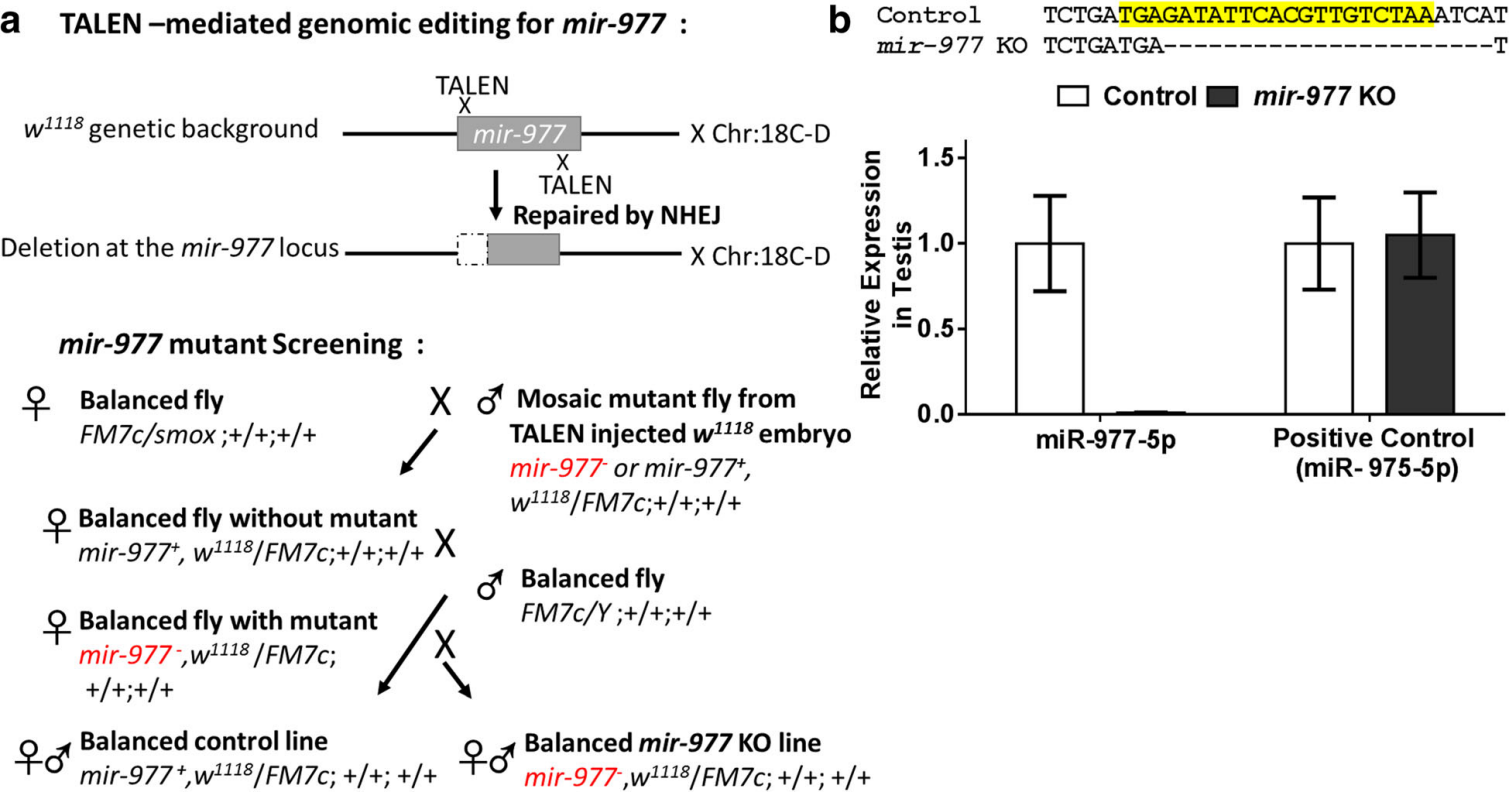
*mir-977* mutant generation*.* **a** Schematic representation of TALEN-mediated mutant generation and screening. **b** *mir-977* deficiency verification. *mir-977* KO results from a 21 bp deletion in its mature (highlighted in yellow) sequence

To achieve a complete deletion of the locus, we used the Transcription activator-like effector nuclease (TALEN) technology [28]. This approach involves designing a nuclease domain that binds next to the *mir-977* mature sequence and makes double-strand breaks in the germline. Imperfect non-homologous end joining repair of these breaks results in deletions (Fig. 2a, upper panel). We used the identical *w*^1118^ white-eyed background for the wildtype and the miRNA knockout (for details, see Additional file 2: Supplementary text 1) and tried our best to avoid the possible off-target effects of TALEN (for details, see Additional file 2: Supplementary text 2). After TALEN injection, we performed a series of crosses to isolate and test candidate mutants (Fig. 2a, lower panel). We succeeded in identifying a 21 bp deletion that spans the mature *mir-977* sequence (Fig. 2b). We then checked *mir-977* expression in testes, the tissue where the wild-type miRNA is exclusively found. We were unable to detect any mature *mir-977-5p* product (Fig. 2b), while expression of the nearby *miR-975* is normal. We therefore conclude that we have successfully constructed a null allele of *mir-977*.

### *mir-977*’s effect on male fitness

Having established a precise *mir-977* deletion in a defined genetic background, we are in a position to assay its fitness effects. Since *mir-977* is expressed exclusively in testes, we focused on measuring male fitness components. The same w1118 background is used in the experiments of the *mir-977* KO flies and in the wildtype control.

#### Male fertility

**Fig. 3:**
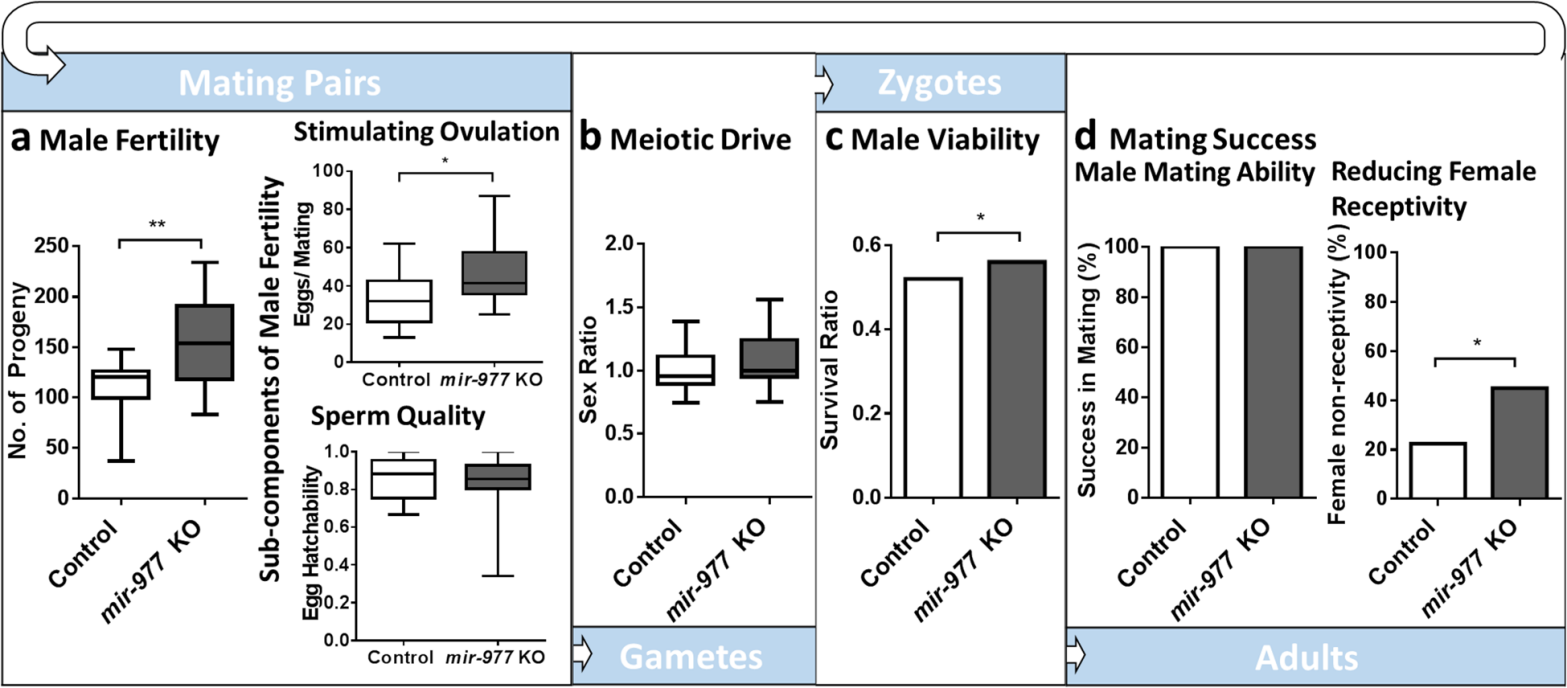
Effect of *mir-977* on male fitness components. **a** Male fertility. Total male fertility is depicted in the left panel. *mir-977* KO have significantly higher fertility compared to the control. On the right panel, we present ovulation stimulation ability (top graph) and sperm quality (bottom). *mir-977* KO results in significantly higher ovulation stimulation ability and a slight decrease (but not significant) in sperm quality. Student’s *t* test *P* values: *: *P* < 0.05; **: *P* < 0.01. **b** Meiotic drive. *mir-977* KO leads to small and statistically insignificant X-chromosome distortion compared to control (Student’s t test *P* > 0.1). **c** Male viability. *mir-977* KO results in a significant improvement in male viability. Binomial test *P* values: *: *P* < 0.05. **d** Mating success. The left panel depicts male mating ability, the right – reduction in female receptivity to subsequent mating. *mir-977* KO results in no changes in mating ability but a significant advantage in reducing female receptivity compared to control males (Chi-square test *P* = 0.03)

We first surveyed total male fertility, a complex phenotype that comprises multiple steps process from successful mating to production of adult offspring. We started by measuring the overall number of adult progeny produced by females of the same genotype mated to control vs *mir-977*^*−*^ males. Strikingly, we observed a significant increase (43.6%, Student’s *t* test *P* = 0.001, *N* = 15) of male fertility in deletion males (Fig. 3a, left panel and Additional file 1: Table S3). It is quite unusual for a gene knockout to outperform the functional gene in such an obvious way. Although the *mir-977* deletion was generated on the same genetic background as the control, it is still possible that an off-site mutation is responsible for the phenotypic effect we observe. To test this, we generated an independent five base-pair deletion in the *mir-977* mature region (listed as *mir-977*KO-2 in Materials and Additional file 1: Figure S1a). We again found an increase in male fertility associated in *mir-977* disruption (35.3%, Student’s *t* test *P* = 0.001, *N* = 15; Additional file 1: Figure S1b and Table S3). We thus conclude that the deletion of *mir-977* itself is indeed responsible for the increase in male fertility we observe.

To further tease apart individual male fertility components (ovulation stimulation and sperm quality), we counted egg production and egg hatchability separately after mating. Deleting *mir-977* increased egg production by 38.4% (Fig. 3a, right panel and Additional file 1: Table S4; Student’s *t* test *P* = 0.04, *N* = 12) compared to control, while slightly, and not statistically significantly, decreasing egg hatchability (Additional file 1: Table S4; 3.1%, Mann Whitney test *P* = 0.74, N = 12). It thus appears that the male fertility benefit of the *mir-977* deficiency is entirely due to an increase in ovulation induction.

#### Meiotic drive

Since *mir-977* is X-linked, it seems plausible that it could exert a fitness effect on the X chromosome at the expense of the Y. We therefore asked whether there is sex-linked meiotic drive associated with this new miRNA [29–31]. To score meiotic drive, we crossed *miR977*^*−*^ or wild type males to *w*^*1118*^*, mir-977*^*+*^ females. We then compared the relative abundance of female and male progeny. Their ratio reflects distortion in sex chromosome transmission. We found a slight, but statistically insignificant, sex ratio distortion effect (3.9%, Student’s *t* test *P* = 0.61, *N* = 20) of *mir-977* deletion (Fig. 3b and Additional file 1: Table S5.

#### Male viability

We next turned to examining the potential effect of knocking out *mir-977* on male viability. We crossed mutant or wild type males to females that bore the same miRNA genotype on one sister X chromosome and a visible balancer chromosome (FM7c, see Methods) on the other. By comparing the abundance of M/Y in the progeny males (Fig. 3c, Additional file 1: Table S6), where M is either *mir-977*^*−*^ or *mir-977*^*+*^, we can measure the effect of the deletion on male viability. The deficiency improved male viability significantly (7.1%, Binomial test, *P* = 0.012). Since *mir-977* expression is confined to males, its deletion should not affect females. To test this, we counted female survival in the same experiment. We found no significant difference between control and *mir-977*^*−*^ females (Binomial test, *P =* 0.48; Additional file 1: Table S6). This again suggests that the effects we observe are the direct consequence of *mir-977* disruption.

#### Mating success

Finally, we assayed male mating ability. It has been well established [32–34] that in addition to success in achieving mating and inducing ovulation, males protect their sperm from competition by subsequently mating males. We already measured ovulation rates and now turn to assessing possible effects of *mir-977* knockout on mating success and sperm competition.

To assay mating success, *w*^*1118*^ females were mated to either control or *mir-977* knockout males (Stage I). After 2.5 days, these mated females were presented with reference males for a second mating (Stage II) and their mating rates at stage I and II were measured (see Methods; Fig. 3d). Our observations show that the *mir-977* deletion did not affect mating success but conferred an advantage over wild type males in reducing female receptivity (43.9% vs 22.5%, Chi-square test *P* = 0.04, *N* = 40). Thus, we again see a beneficial effect of *mir-977* elimination on male mating success.

## Discussion

To investigate the potential fates of genes that have originated de novo relatively recently in evolutionary time, we focused on *mir-977*. This microRNA originated not long prior to the origin of the *Drosophila* clade 60 Myrs ago. While it then evolved conservatively on the deeper branches, it has recently been lost in the *D. pseudoobscura* lineage and has been evolving fast in the *D. melanogaster* subgroup. To further probe the possible fate of *mir-977* in *D. melanogaster*, we created a complete knockout of this gene and assayed the effects of the deletion on male fitness. Interestingly, we see a consistent increase in performance of the deficiency across male fitness components, most notably fertility, viability and prevention of female re-mating.

Fitness effects of a mutation can depend on the environmental context [35]. Since it is not feasible to assay all relevant conditions, particularly because we do not know all variables encountered by flies in the wild, we cannot rule out the possibility that loss of *mir-977* can be deleterious under some circumstances. However, given the much faster evolution of this gene in younger *Drosophila* lineages and its complete loss in *D. pseudoobscura*, impending loss of *mir-977* in *D. melanogaster* appears to be a reasonable expectation. If so, our results suggest that rather than becoming non-functional, this miRNA has turned actively deleterious to male reproductive functions (For possible mechanisms, see Additional file 2: Supplementary text 3). In this situation, natural selection is ready to eliminate *mir-977* when the right mutant arrives.

Why then has *mir-977* not been removed? First, the mechanism most conducive for eliminating a miRNA gene would be small deletions (Single base pair substitutions are usually too weak). However, within the tight cluster of miRNA genes in the neighborhood of *mir-977*, most such deletions may delete (parts of) the neighboring genes and are thus deleterious. Second, given the advantage of the right deletion, its spread in the population would be rapid and the polymorphism of a null *mir-977* mutation would last only briefly. In contrast, an observed polymorphism is more likely the indication of a fitness-neutral mutation slowly drifting in the population [36]. From the polymorphism database (*Drosophila* Population Genomics Project [37]), we did not find evidence for *mir-977* polymorphism. Third, this gene has indeed been eliminated at least twice independently in *Drosophila* (*D. pseudoobscura* and *D. erecta*, see Fig. 1b). In short, a deleterious *mir-977* gene would be retained for a while in the population until the right deletion happens. Then, it would be lost very quickly from the population. Taken together, our results provide insights into life cycles of de novo genes (Additional file 1: Figure S2).

Our results suggest a general approach to studying novel gene death. Clearly, sequence evolution alone is insufficient to predict gene fate. However, adding experimental results can yield additional insights. What has happened to shift the fitness contribution of *mir-977* to the testes transcriptome? Ecological factors can have changed, rendering its effects deleterious in the new environments. Alternatively, or perhaps additionally, the transcriptional regulatory network topology may have been re-arranged during evolution. Detailed transcriptome studies in multiple species with *mir-977* present or absent may answer these questions.

## Conclusion

The constant rises and falls of de novo genes suggest that de novo genes do play a role in adaptation. Interestingly, the transient nature of the adaptive functions almost guarantee the controversy surrounding them.

## Methods

### Fly stocks and *mir-977* mutant generation

Flies were raised in standard media at 25 °C under a 12:12 h light/dark cycle. Stocks (*w*^*1118*^ and *FM7c* (NO.12246)) were obtained from the Bloomington Stock center. The reference line for mating success assays was *w*^*1118*^/Y; *miniwhite-UASeGFP*/*miniwhite-UASeGFP*, a red-eyed strain with an insertion of *mini-white* at the 51D position on chromosome 2 via the PhiC31 site-specific chromosomal integration system. The crossing scheme for *mir-977* mutant generation is shown in Fig. 2a. miRNA mutants were detected by PCR and verified by qRT-PCR (Primers are shown in Additional file 1: Table S8). To make sure no exchange of material between the balancer and the balanced chromosome, all lines have been subjected to PCR to confirm the correct genotype.

### *mir-977* KO verification by qRT-PCR

Three batches of 30–50 testes each were collected independently as biological replicates for qRT-PCR assays. Total RNA was extracted using the Ambion TRIzol® Reagent (code No. 15596018). qRT-PCR of miRNAs was conducted using stem-loop reverse transcription [38] followed by TaqMan PCR analysis using the miRNA UPL (Roche Diagnostics) probe assay protocol [39]. Relative miRNA expression levels were estimated using the 2^−ΔΔCT^method [40]. 2S RNA was used as the endogenous control.

### Sequence and expression analysis of *mir-977* across species

Mature *mir-977* sequences were retrieved from the current Release 21 of miRBase (http://www.mirbase.org) [41]. Secondary structures of miRNA precursors were predicted using RNA-fold (http://rna.tbi.univie.ac.at/cgi-bin/RNAWebSuite/RNAfold.cgi) [42].

Expression analyses were based on miRNA deep sequence data, using small RNA libraries from testes (except for *D. erecta*, since only male whole-body data are available for this species). The data were retrieved from the GEO database (http://www.ncbi.nlm.nih.gov/geo/, accession numbers GSM909277 for *D. melanogaster-* Oregon R-1,GSM 909278 for *D. melanogaster-*Oregon R-2, GSM 548591 for *D. melanogaster-* hs-Penlope, GSM 548589 for *D. melanogaster-*A1,GSM 548584 for *D.melanogaster-*yw67c23(2),GSM1165053 for *D. simulans*, GSM1357621 for *D. erecta*, and GSM5486109 for *D. virilis*). miRNA precursors in *D. melanogaster* were retrieved from miRBase [41]. The orthologous precursors from *D. simulans, D. erecta*, and *D. virilis* were obtained from [43]. Mature miRNA sequences were retrieved from the miRBase. Missing annotations of miR-972 cluster members in *D. simulans, D. erecta*, and *D.virilis* were supplemented with data from [10] and miRdeep2 prediction [44]. miRNA mature sequence expression was quantified using the quantifier.pl script from miRdeep2 (version 2.0.0.7), using all reads mappable to the mature sequences and scaling as Reads Per Million (RPM) in each library. miRNA expression is the sum of two mature products from a given precursor.

### Fitness component assays

#### Male fertility

To survey male fertility, each three to five-day-old *mir-977* KO or control male was mated to three to five-day-old virgin wild-type females for two days. The progeny for each mating was counted to assay male fertility. Twenty replicate crosses were set up for each genotype.

To survey the sub-components (ovulation stimulation and sperm quality) of male fertility, each three to five-day-old *mir-977*^*−*^ or control male was mated to one virgin wild-type female for two days. We then counted the number of eggs and progeny (1st larvae) to estimate egg production (as male’s ability to stimulate ovulation) and egg hatchability (as sperm quality). Twelve replicate crosses were set up for each genotype (Raw data are shown in Additional file 3).

#### Meiotic drive

To survey the distortion in sex chromosome transmission, we crossed hemizygous *mir-977* KO or control males (M/Y) to *w*^*1118*^ females (*w*^*1118*^*/w*^*1118*^). In the F1 progeny for each cross, the ratio of *w*^*1118*^*/*M females to *w*^*1118*^/Y males reflects the distortion in sex chromosome transmission of the *mir-977*^*+*^ or *mir-977*^*−*^ chromosome. Twenty replicate crosses were set up for each genotype (Raw data are shown in Additional file 3).

#### Viability

Viability assays followed the protocol described in Chen et al. [27], we crossed balanced *mir-977* KO or control females (FM7c/M) to hemizygous mutant or control males (M/Y). The number of M/Y in F1 male progeny in these crosses reflects male viability of control or *mir-977* KO males compared to balancer males. As a confirmatory experiment, survival of M/M females reflects female viability of control or *mir-977* KO females compared to balancer females. We counted 350 to 500 offspring of each sex and genotype in this assay.

#### Mating success

To survey mating success (male ability to successfully mate and to repress female re-mating), each *w*^*1118*^ female was mated to either a control or a *mir-977* KO male (Stage I); after 2.5 days, these mated females were presented with reference males for a second mating (Stage II). Both control and *mir-977* KO males were white-eyed but reference males were red-eyed. Mating rates at stages I and II can then be inferred from the eye color of F1 progeny of each mating. F0 females with white-eyed progeny suggest successful fertilization at stage I; F0 females with both white-eyed and red-eyed progeny suggest successful fertilization at stage II. Stage I and II mating rates reflect male mating ability and female receptivity. Forty replicate crosses were set up for each genotype.

## Additional files

Additional file 1: **Figure S1.** Independent deletion confirms male fertility increase due to *mir-977* deletion. **Figure S2.** Lifecycle of new gene. **Table S1.** *mir-977* expression in Drosophila species. **Table S2.** Expression variation of *mir-977* in testes across 5 lines of *D. melanogaster*. **Table S3.** Male fertility of *mir-977* KO. **Table S4.** Stimulating ovulation and sperm quality of *mir-977* KO. **Table S5.** Meiotic drive of *mir-977* KO. **Table S6.** Viability of *mir-977* KO. **Table S7.** Mating success of *mir-977* KO. **Table S8.** Primers used in this study. **Table S9.** TALEN pairs design for *mir-977*. (PPTX 277 kb)

Additional file 2: Supplementary Text 1.The choice of genetic background. Supplementary Text 2. Discussion about the off-target effect. Supplementary Text 3. Possible mechanism of *mir-977*’s phenotypic effect. (PDF 414 kb)

Additional file 3: Raw data of male fitness components for *mir-977* KO. Detail components: male fertility (related to Fig. 3a and Additional file 1: Figure S1b), stimulating ovulation (related to Fig. 3a), sperm quality (related to Fig. 3a), meiotic drive (related to Fig. 3b). (XLSX 12 kb)

## Abbreviations

*D. mel*: *D. melanogaster*
*D. pse*: *D. pseudoobscura*
*D. sim*: *D. simulans*
*D. vir*: *D. virilis*
*D.ere*: *D. erecta*
KO: Knockout
*mir-977*^−^: *mir-977* knockout line
*mir-977*^*+*^: control, the wildtype strain
NHEJ: Non-homologous end joining
RPM: Reads Per Million
Small RNA-seq: microRNA high-throughput sequencing
TALEN: Transcription activator-like effector nuclease technology
