# Supplementary material for: On the possibility of death of new genes – evidence from the deletion of de novo microRNAs
Source: BMC Genomics. 2018 May 23;19:388. doi: 10.1186/s12864-018-4755-1 (PMC5966946; doi:10.1186/s12864-018-4755-1)
Supplement: Supplementary file 1 — Figure S1. Independent deletion confirms male fertility increase due to mir-977 deletion. Figure S2. Lifecycle of new gene. Table S1. mir-977 expression in Drosophila species. Table S2. Expression variation of mir-977 in testes across 5 lines of D. melanogaster. Table S3. Male fertility of mir-977 KO. Table S4. Stimulating ovulation and sperm quality of mir-977 KO. Table S5. Meiotic drive of mir-977 KO. Table S6. Viability of mir-977 KO. Table S7. Mating success of mir-977 KO. Table S8. Primers used in this study. Table S9. TALEN pairs design for mir-977. (PPTX 277 kb) [file 12864_2018_4755_MOESM1_ESM.pptx]

## Slide 1
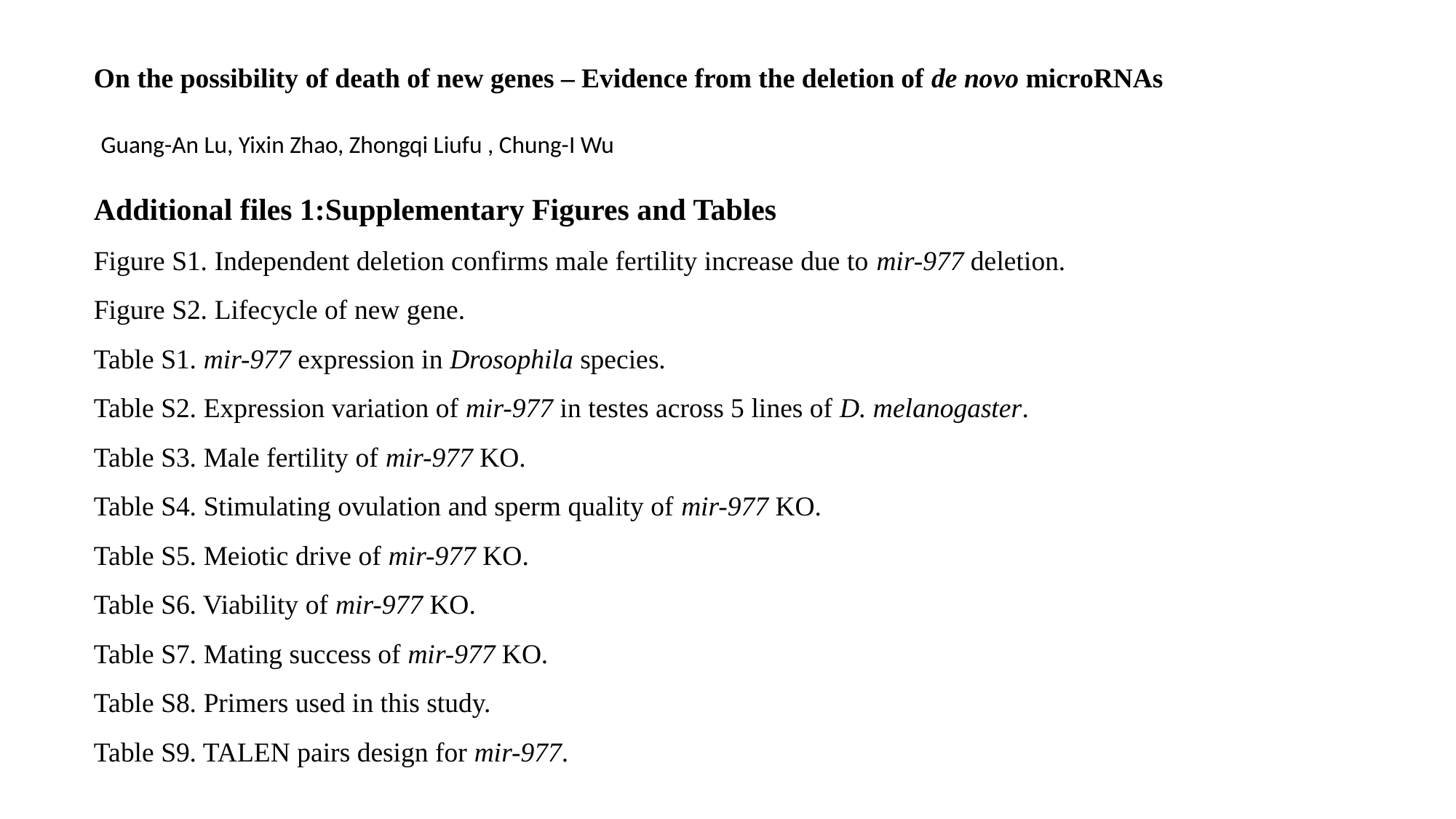

On the possibility of death of new genes – Evidence from the deletion of de novo microRNAs
 Guang-An Lu, Yixin Zhao, Zhongqi Liufu , Chung-I Wu
Additional files 1:Supplementary Figures and Tables
Figure S1. Independent deletion confirms male fertility increase due to mir-977 deletion.
Figure S2. Lifecycle of new gene.
Table S1. mir-977 expression in Drosophila species.
Table S2. Expression variation of mir-977 in testes across 5 lines of D. melanogaster.
Table S3. Male fertility of mir-977 KO.
Table S4. Stimulating ovulation and sperm quality of mir-977 KO.
Table S5. Meiotic drive of mir-977 KO.
Table S6. Viability of mir-977 KO.
Table S7. Mating success of mir-977 KO.
Table S8. Primers used in this study.
Table S9. TALEN pairs design for mir-977.

## Slide 2
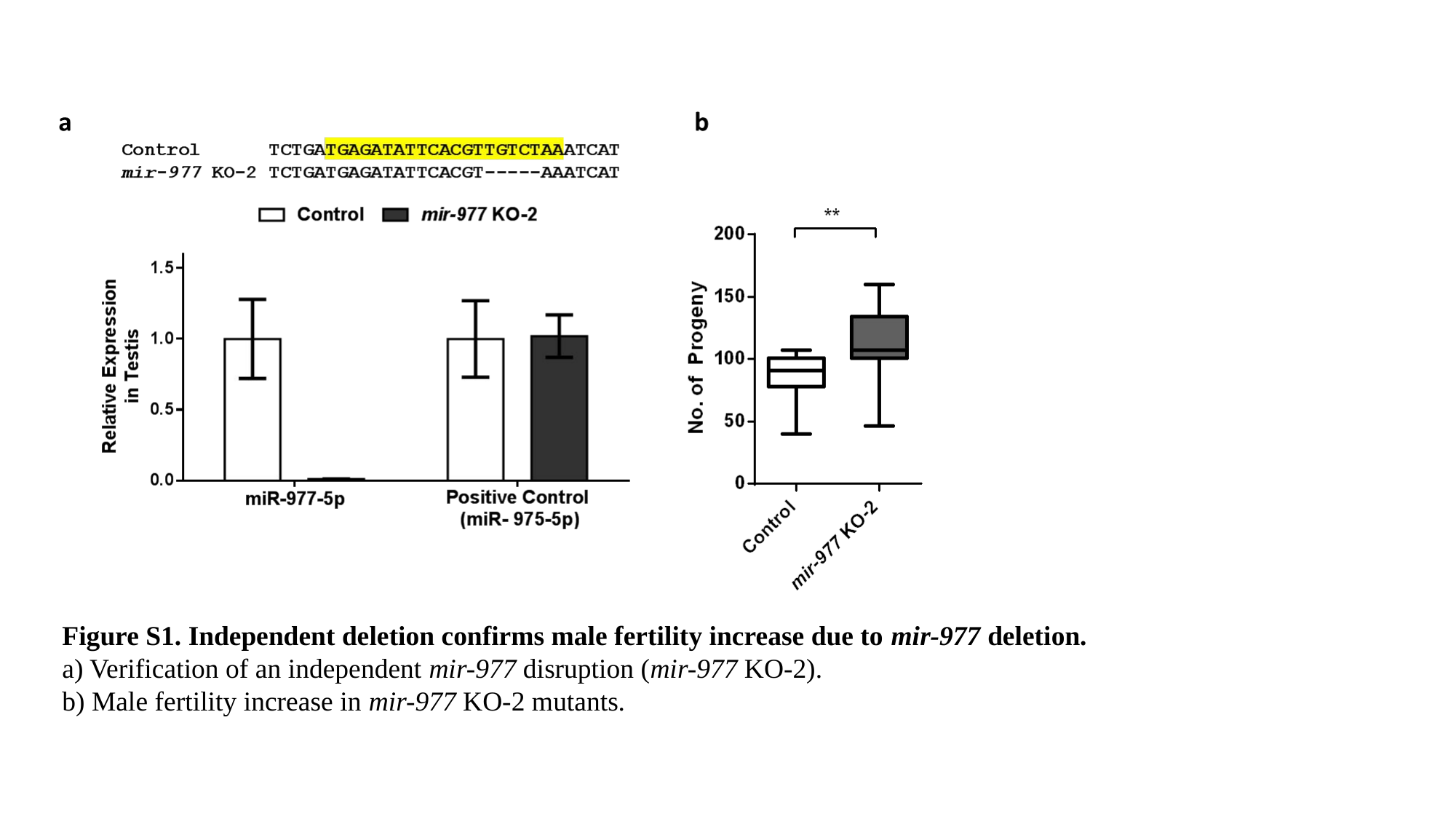

Figure S1. Independent deletion confirms male fertility increase due to mir-977 deletion.
a) Verification of an independent mir-977 disruption (mir-977 KO-2).
b) Male fertility increase in mir-977 KO-2 mutants.

## Slide 3
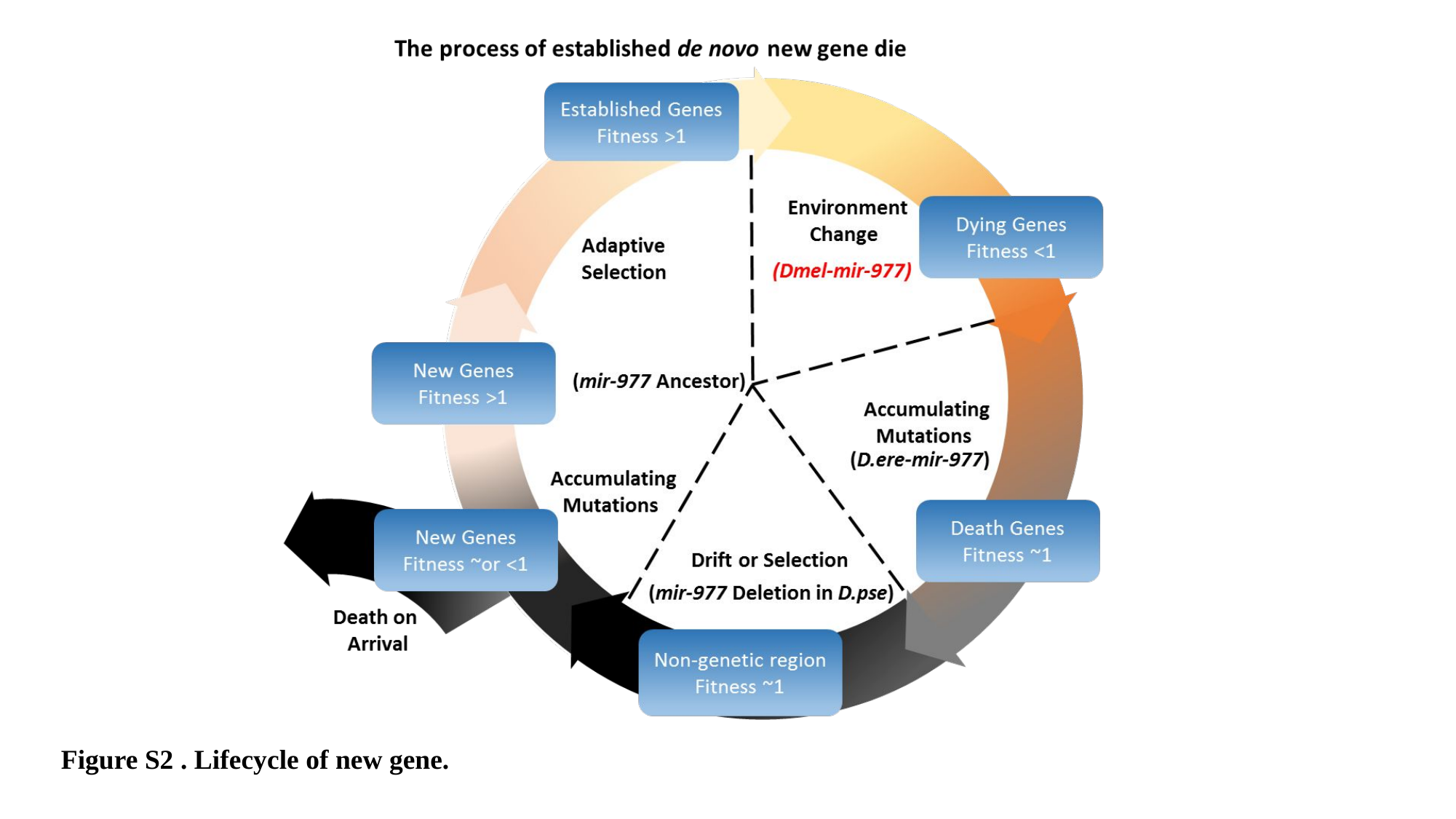

Figure S2 . Lifecycle of new gene.

## Slide 4
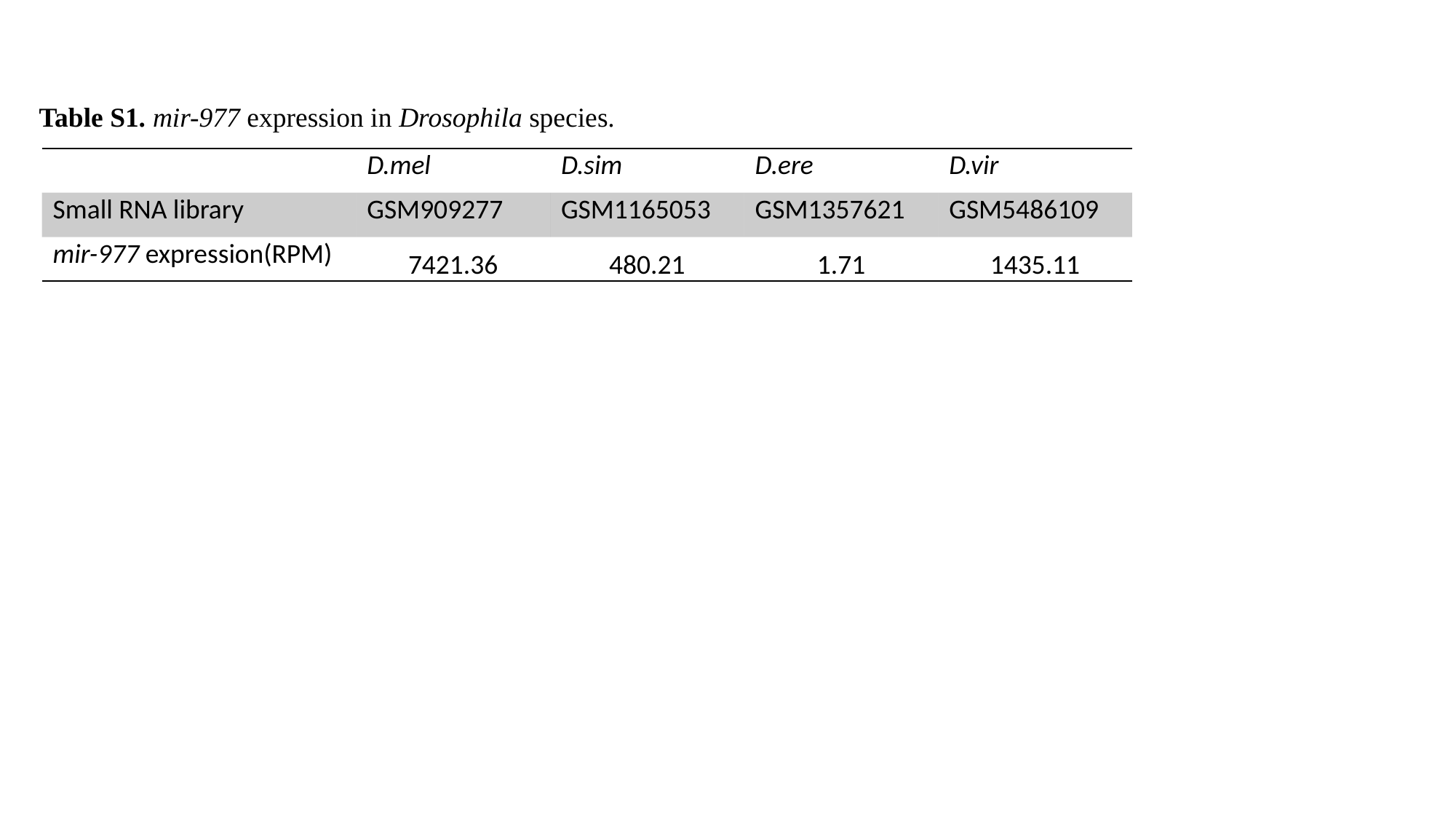

Table S1. mir-977 expression in Drosophila species.
| | D.mel | D.sim | D.ere | D.vir |
| --- | --- | --- | --- | --- |
| Small RNA library | GSM909277 | GSM1165053 | GSM1357621 | GSM5486109 |
| mir-977 expression(RPM) | 7421.36 | 480.21 | 1.71 | 1435.11 |

## Slide 5
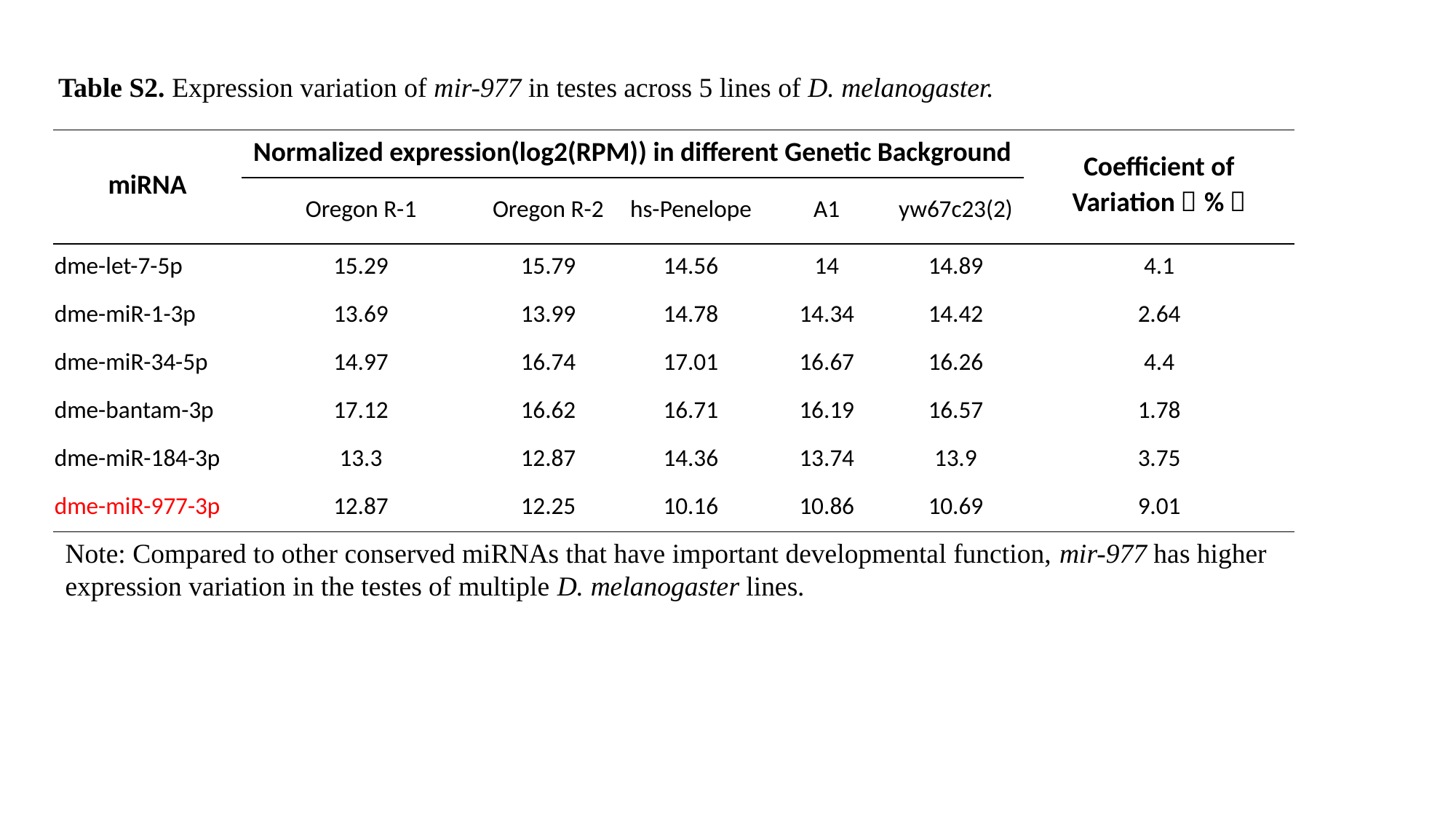

Table S2. Expression variation of mir-977 in testes across 5 lines of D. melanogaster.
| miRNA | Normalized expression(log2(RPM)) in different Genetic Background | | | | | Coefficient of Variation（%） |
| --- | --- | --- | --- | --- | --- | --- |
| | Oregon R-1 | Oregon R-2 | hs-Penelope | A1 | yw67c23(2) | |
| dme-let-7-5p | 15.29 | 15.79 | 14.56 | 14 | 14.89 | 4.1 |
| dme-miR-1-3p | 13.69 | 13.99 | 14.78 | 14.34 | 14.42 | 2.64 |
| dme-miR-34-5p | 14.97 | 16.74 | 17.01 | 16.67 | 16.26 | 4.4 |
| dme-bantam-3p | 17.12 | 16.62 | 16.71 | 16.19 | 16.57 | 1.78 |
| dme-miR-184-3p | 13.3 | 12.87 | 14.36 | 13.74 | 13.9 | 3.75 |
| dme-miR-977-3p | 12.87 | 12.25 | 10.16 | 10.86 | 10.69 | 9.01 |
Note: Compared to other conserved miRNAs that have important developmental function, mir-977 has higher
expression variation in the testes of multiple D. melanogaster lines.

## Slide 6
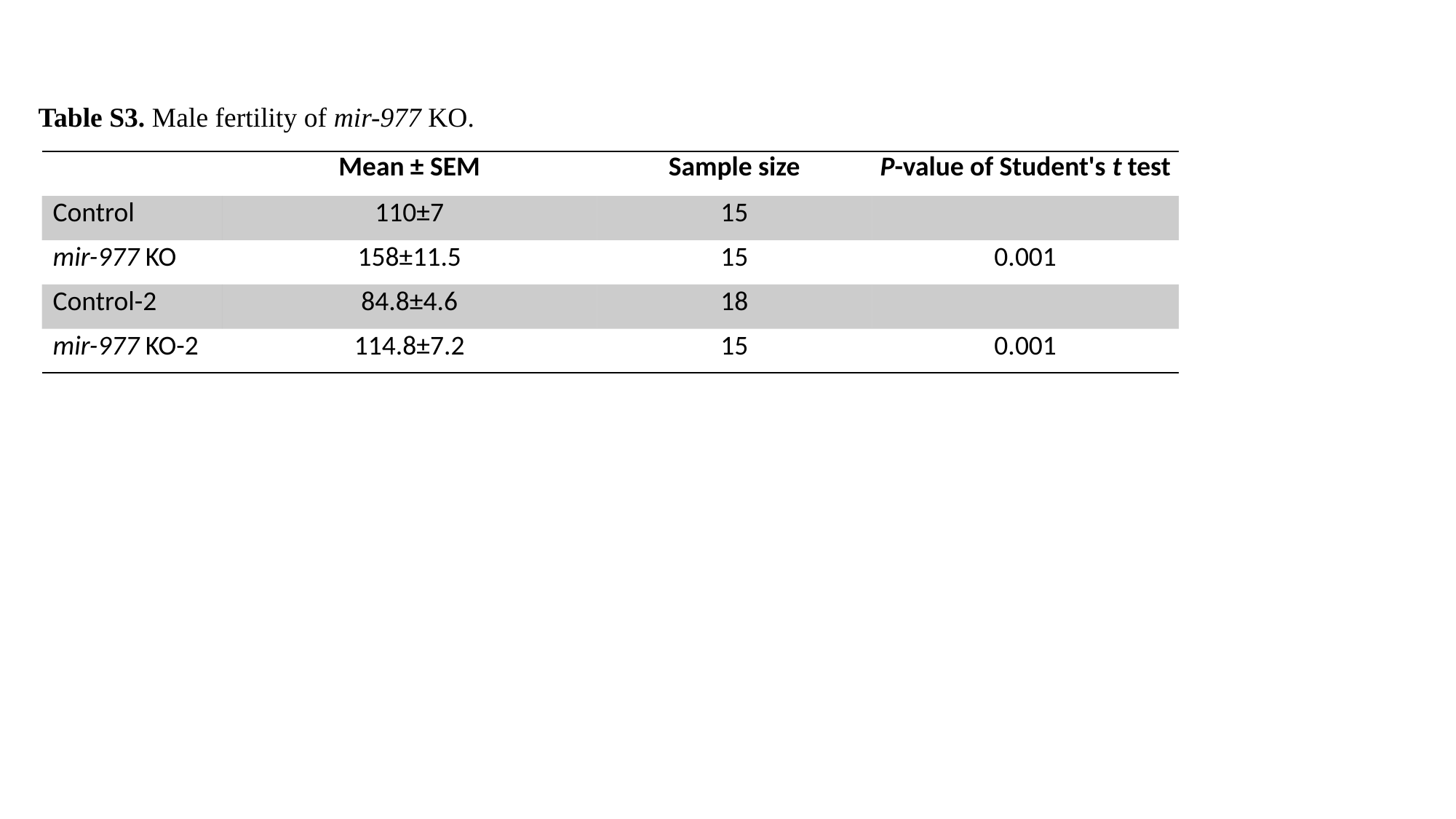

Table S3. Male fertility of mir-977 KO.
| | Mean ± SEM | Sample size | P-value of Student's t test |
| --- | --- | --- | --- |
| Control | 110±7 | 15 | |
| mir-977 KO | 158±11.5 | 15 | 0.001 |
| Control-2 | 84.8±4.6 | 18 | |
| mir-977 KO-2 | 114.8±7.2 | 15 | 0.001 |

## Slide 7
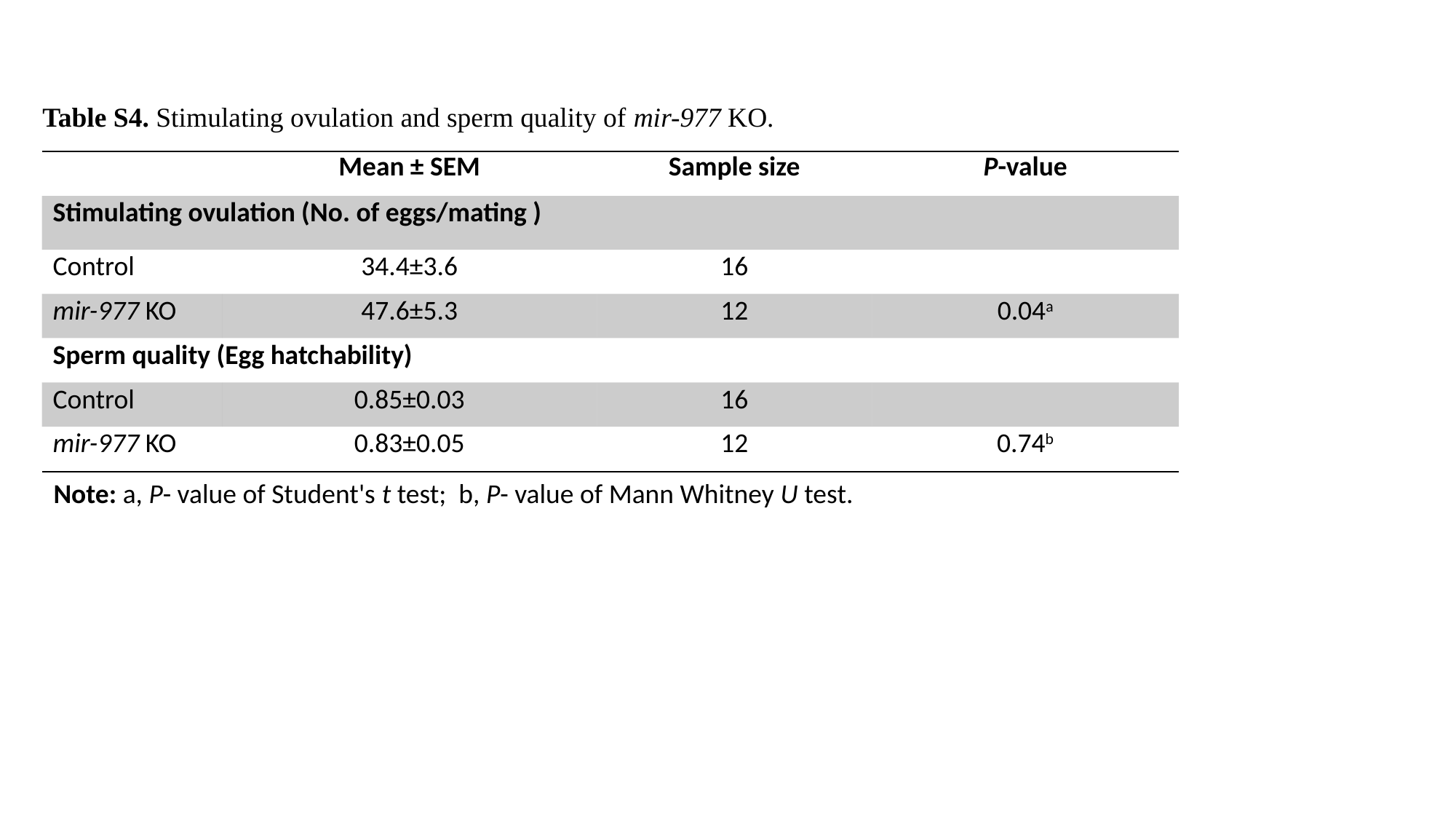

Table S4. Stimulating ovulation and sperm quality of mir-977 KO.
| | Mean ± SEM | Sample size | P-value |
| --- | --- | --- | --- |
| Stimulating ovulation (No. of eggs/mating ) | | | |
| Control | 34.4±3.6 | 16 | |
| mir-977 KO | 47.6±5.3 | 12 | 0.04a |
| Sperm quality (Egg hatchability) | | | |
| Control | 0.85±0.03 | 16 | |
| mir-977 KO | 0.83±0.05 | 12 | 0.74b |
Note: a, P- value of Student's t test; b, P- value of Mann Whitney U test.

## Slide 8
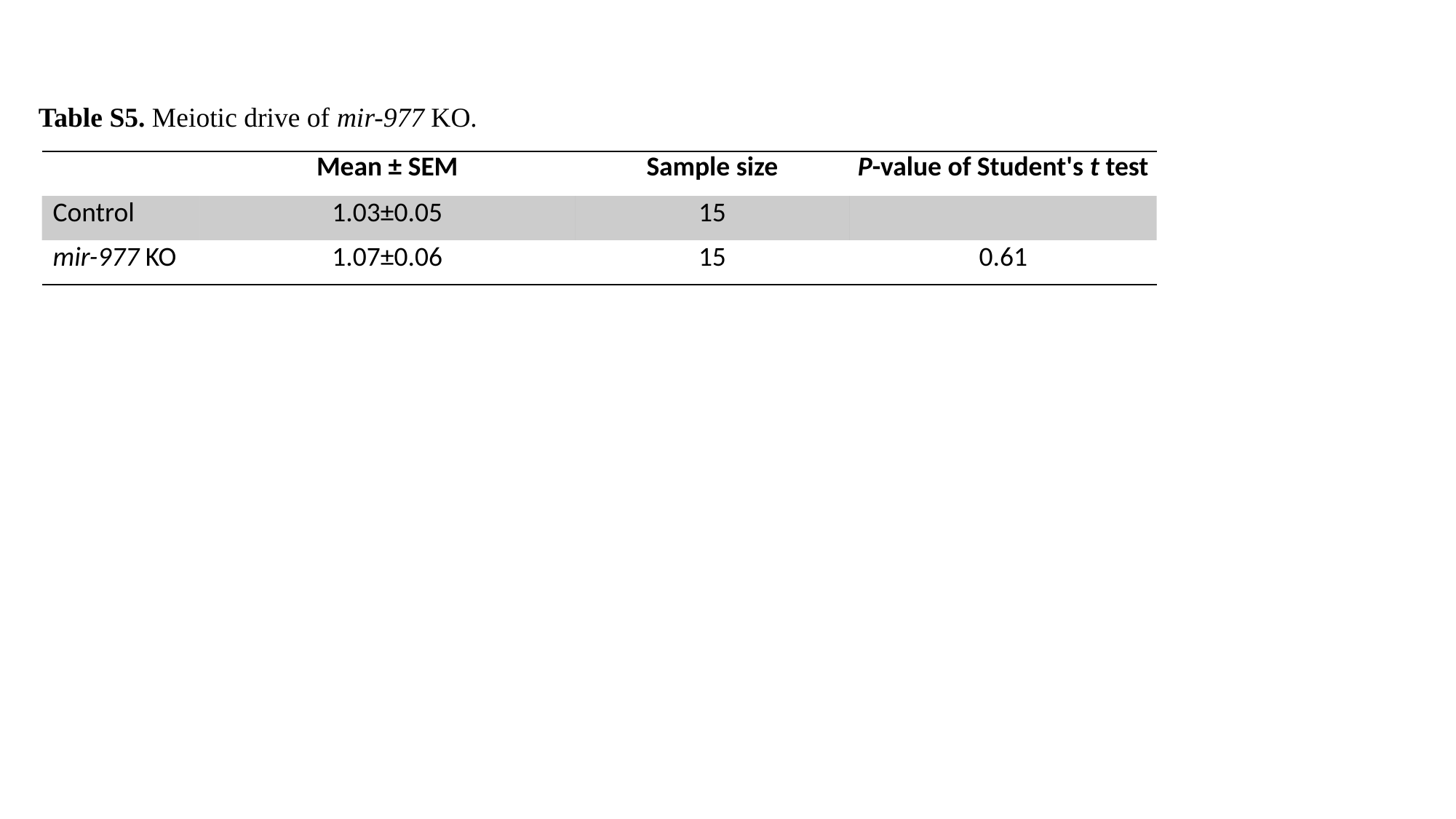

Table S5. Meiotic drive of mir-977 KO.
| | Mean ± SEM | Sample size | P-value of Student's t test |
| --- | --- | --- | --- |
| Control | 1.03±0.05 | 15 | |
| mir-977 KO | 1.07±0.06 | 15 | 0.61 |

## Slide 9
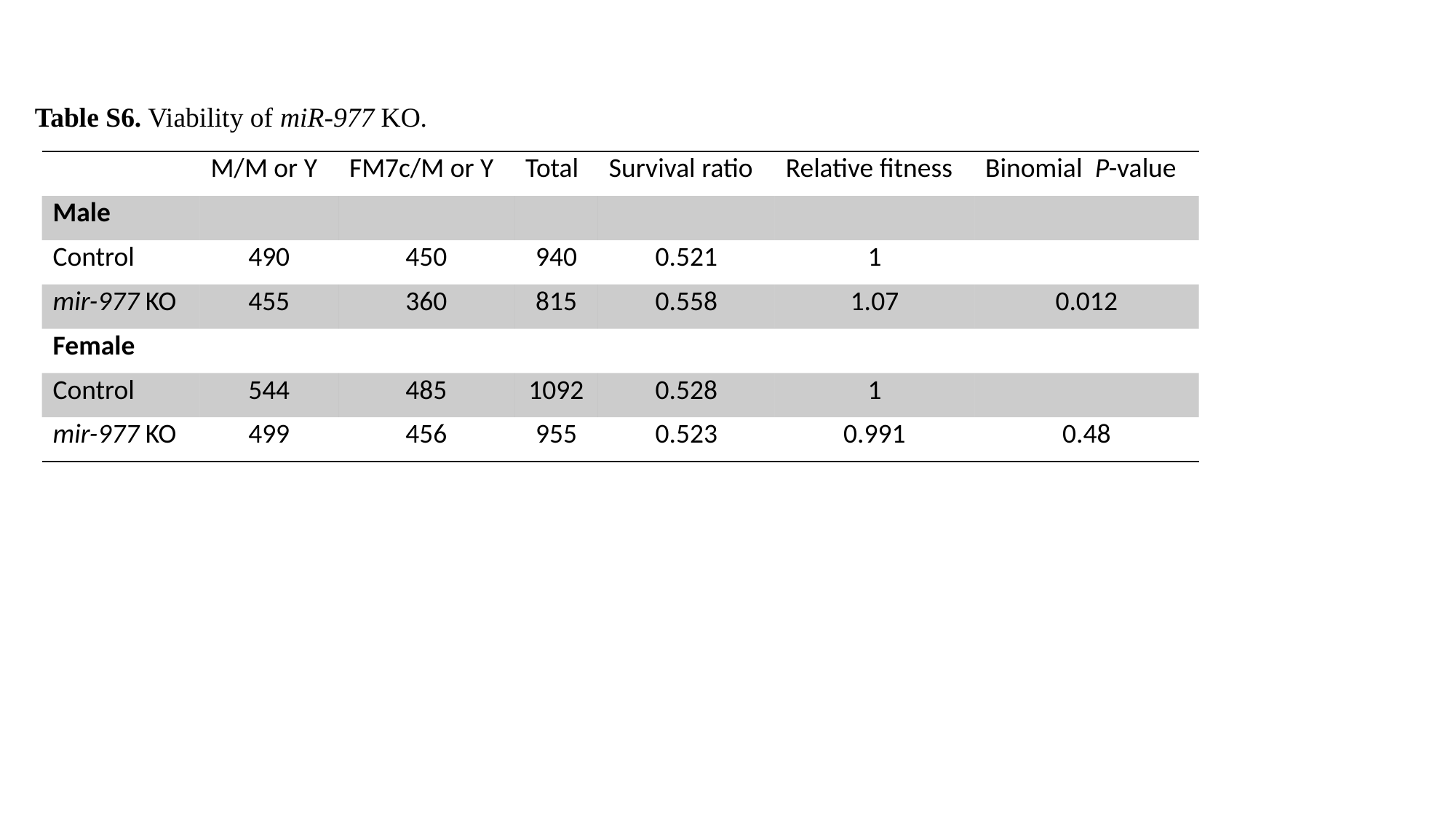

Table S6. Viability of miR-977 KO.
| | M/M or Y | FM7c/M or Y | Total | Survival ratio | Relative fitness | Binomial P-value |
| --- | --- | --- | --- | --- | --- | --- |
| Male | | | | | | |
| Control | 490 | 450 | 940 | 0.521 | 1 | |
| mir-977 KO | 455 | 360 | 815 | 0.558 | 1.07 | 0.012 |
| Female | | | | | | |
| Control | 544 | 485 | 1092 | 0.528 | 1 | |
| mir-977 KO | 499 | 456 | 955 | 0.523 | 0.991 | 0.48 |

## Slide 10
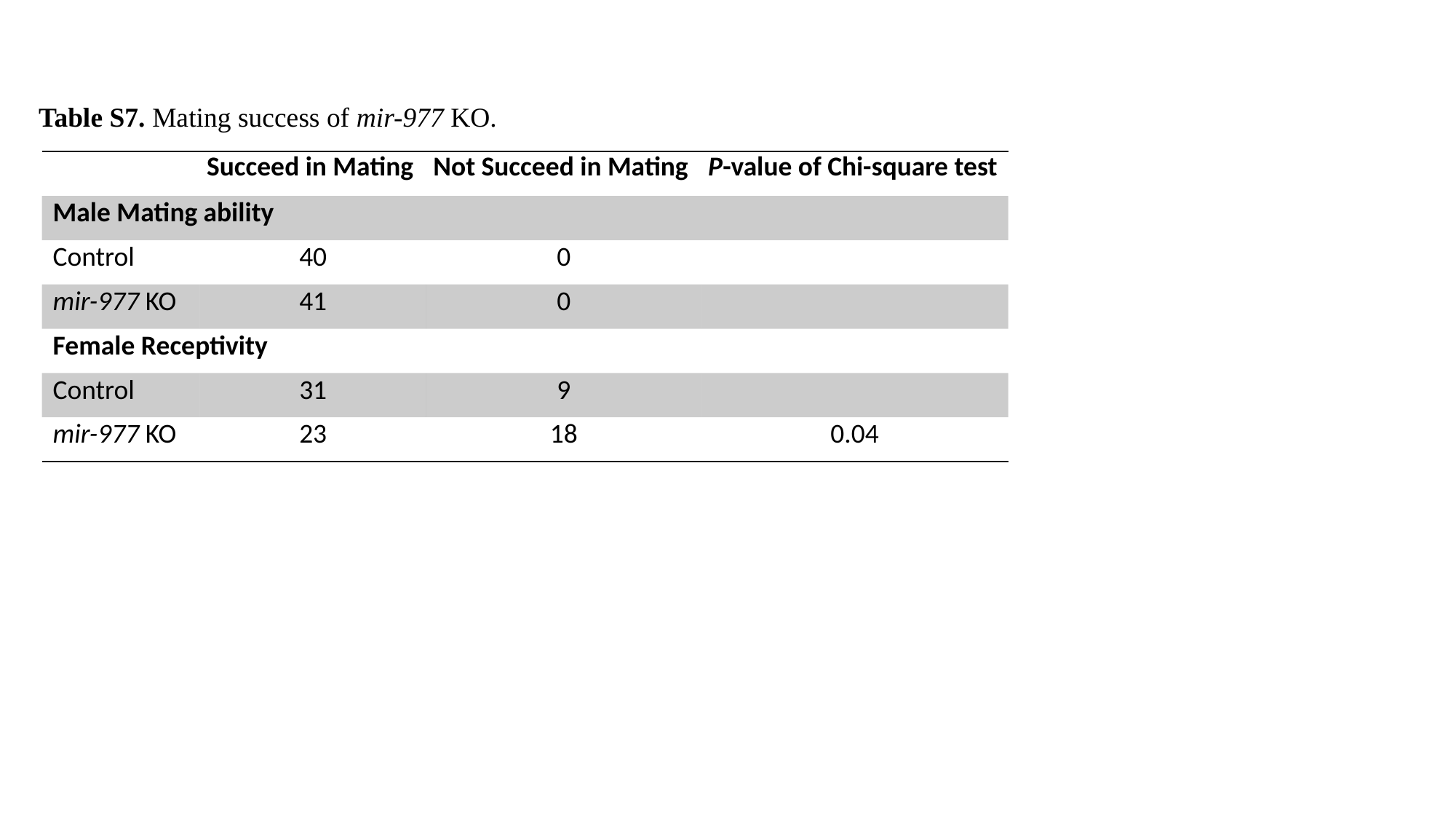

Table S7. Mating success of mir-977 KO.
| | Succeed in Mating | Not Succeed in Mating | P-value of Chi-square test |
| --- | --- | --- | --- |
| Male Mating ability | | | |
| Control | 40 | 0 | |
| mir-977 KO | 41 | 0 | |
| Female Receptivity | | | |
| Control | 31 | 9 | |
| mir-977 KO | 23 | 18 | 0.04 |

## Slide 11
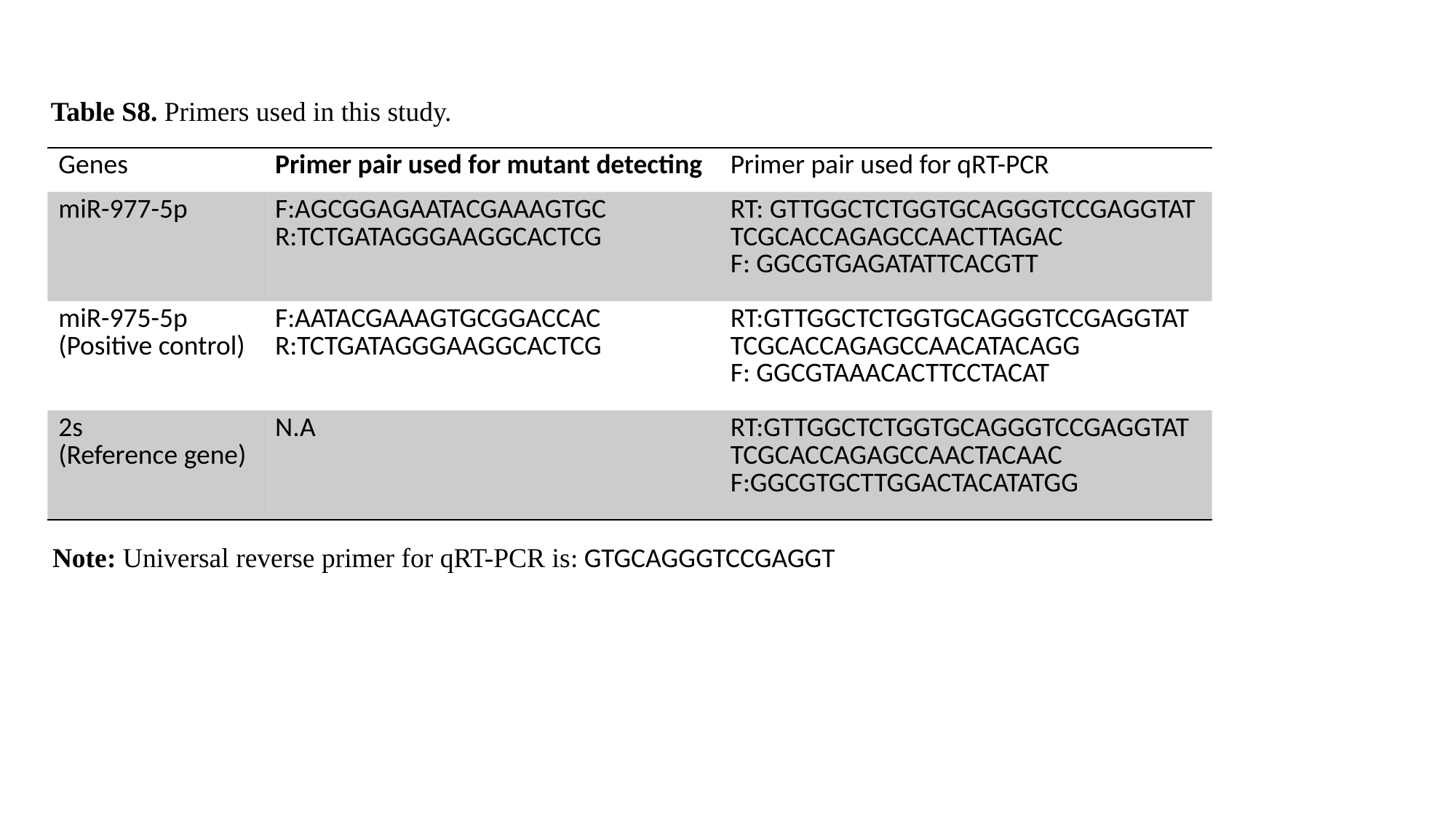

Table S8. Primers used in this study.
| Genes | Primer pair used for mutant detecting | Primer pair used for qRT-PCR |
| --- | --- | --- |
| miR-977-5p | F:AGCGGAGAATACGAAAGTGC R:TCTGATAGGGAAGGCACTCG | RT: GTTGGCTCTGGTGCAGGGTCCGAGGTAT TCGCACCAGAGCCAACTTAGAC F: GGCGTGAGATATTCACGTT |
| miR-975-5p (Positive control) | F:AATACGAAAGTGCGGACCAC R:TCTGATAGGGAAGGCACTCG | RT:GTTGGCTCTGGTGCAGGGTCCGAGGTATTCGCACCAGAGCCAACATACAGG F: GGCGTAAACACTTCCTACAT |
| 2s (Reference gene) | N.A | RT:GTTGGCTCTGGTGCAGGGTCCGAGGTATTCGCACCAGAGCCAACTACAAC F:GGCGTGCTTGGACTACATATGG |
Note: Universal reverse primer for qRT-PCR is: GTGCAGGGTCCGAGGT

## Slide 12
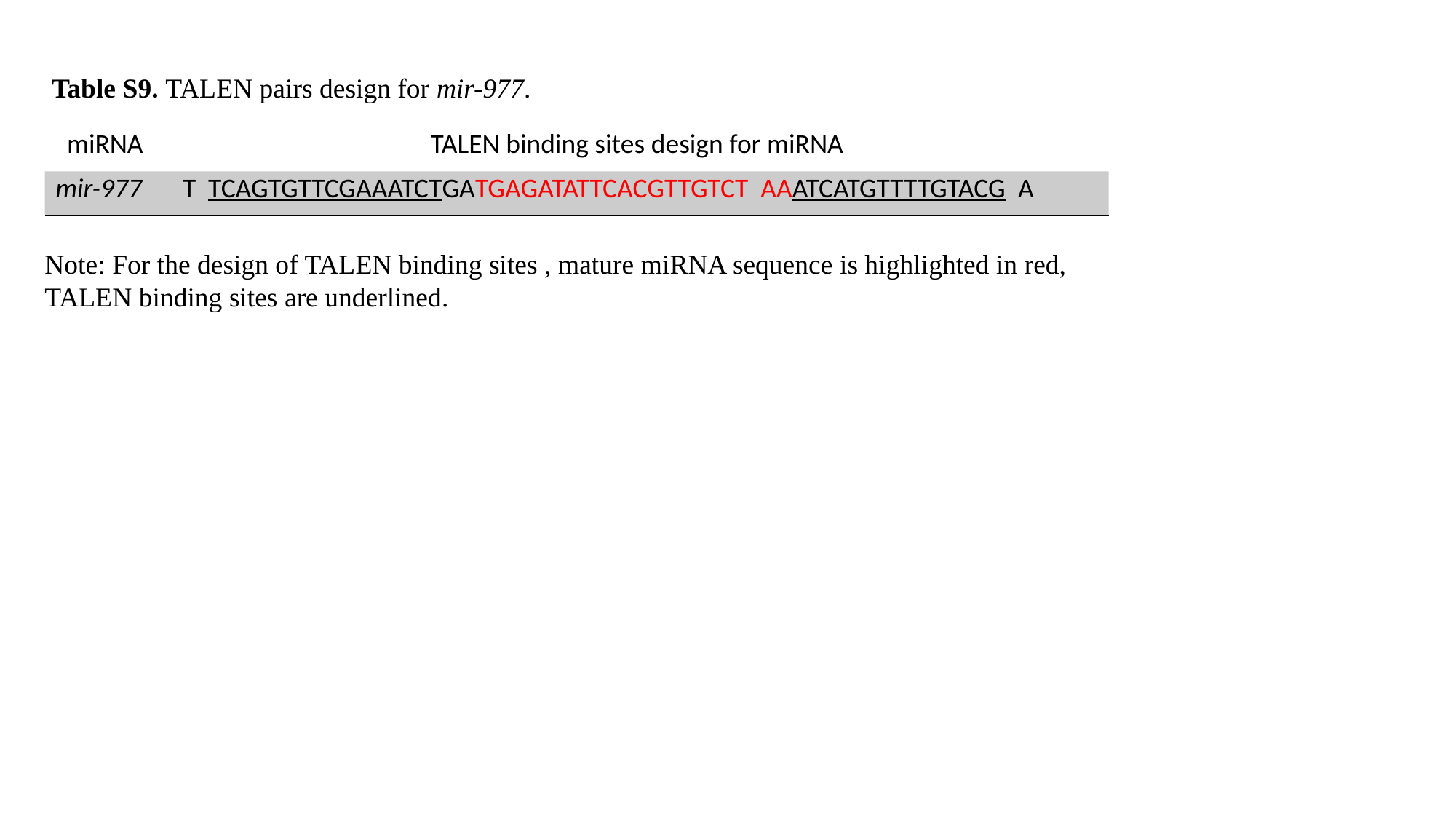

Table S9. TALEN pairs design for mir-977.
| miRNA | TALEN binding sites design for miRNA |
| --- | --- |
| mir-977 | T TCAGTGTTCGAAATCTGATGAGATATTCACGTTGTCT AAATCATGTTTTGTACG A |
Note: For the design of TALEN binding sites , mature miRNA sequence is highlighted in red,
TALEN binding sites are underlined.
