# Supplementary material for: On the possibility of death of new genes – evidence from the deletion of de novo microRNAs
Source: BMC Genomics. 2018 May 23;19:388. doi: 10.1186/s12864-018-4755-1 (PMC5966946; doi:10.1186/s12864-018-4755-1)
Supplement: Supplementary file 2 — Supplementary Text 1.The choice of genetic background. Supplementary Text 2. Discussion about the off-target effect. Supplementary Text 3. Possible mechanism of mir-977’s phenotypic effect. (PDF 414 kb) [file 12864_2018_4755_MOESM2_ESM.pdf]

On the possibility of death of new genes – Evidence from the deletion of *de novo* microRNAs

Guang-An Lu, Yixin Zhao, Zhongqi Liufu , Chung-I Wu

Additional files 2: Supplementary Texts

Supplementary Text 1.The choice of genetic background.

Supplementary Text 2.Discussion about the off-target effect.

Supplementary Text 3. Possible mechanism of *mir-977*'s phenotypic effect.

Supplementary Text 1.The choice of genetic background.

While we use the identical *w1118* white-eyed background for the wildtype and the miRNA knockout, there is a concern in this application due to a change in the color of the testis as well as a slight reduction in size. For two reasons, we (and others) did not switch the measurements to the wildtype background. First, if we try to replace the white-eyed allele (which is necessary to monitor the X chromosome during the construction), the parallel replacements are unlikely to yield the identical background for the wildtype and KO lines. Second, we have used the *w1118* background in our previous publications [1-3], many pertaining to male reproduction, and have not encountered warning signs about its suitability in such experiments.

Nevertheless, caveats need to be stated that certain aspects of male reproduction might yield artificial results in the *w1118* background.

References:

1. Liufu Z, Zhao Y, Guo L, Miao G, Xiao J, Lyu Y, Chen Y, Shi S, Tang T, Wu CI: **Redundant and incoherent regulations of multiple phenotypes suggest microRNAs' role in stability control**. *Genome Research* 2017, **27**: 1665-1673.
2. Sun S, Ting C, Wu C: **The Normal Function of a Speciation Gene, Odysseus, and Its Hybrid Sterility Effect**. *Science* 2004, **305**(5680):81-83.
3. Greenberg AJ, Moran JR, Coyne JA, Wu CI: **Ecological adaptation during incipient speciation revealed by precise gene replacement**. 2003, **302**(5651):1754-1757.

Supplementary Text 2.Discussion about the off-target effect.

For the possible off-target effects. While such effects can sometimes be problematic, the situation here is rather different. Note that the deletion has a fitness-enhancing effect. It would seem rather unlikely that off-target effects, if present in this case, would give the KO lines a higher fitness.

On the off-target effects: While the main argument is that off-target effects are very unlikely to INCREASE the fitness, as observed, we also provide additional discussions on such effects as supplement. To our knowledge, there are two kinds of off-target effect and we have tried our best to avoid them.

1) Assuming there is no mis-match of TALEN binding, off target effect stems from specifically binding of TALEN to other genome regions besides the locus of *mir-977*. Such effect has been considered when design the TALEN pairs of *mir-977*. Here, we can open our TALEN pairs design (TableS5) and this pairs just target the locus of *mir-977* only.

2) Assuming there exists mis-match of TALEN bindings, off-target effect stems from randomly binding of TALEN pairs to other genome regions besides the locus of *mir-977*. To exclude this possibility and confirm our results, we first use two independent knockout to present consist increase pattern in male fertility, on the other, in the viability assay, we use a couple of components of female fitness as a control. Since *mir-977* is just found in male testis, one would expect female fitness should not be affected, such results have been showed in Table S2.

Supplementary Text 3. Possible mechanism of *mir-977*'s phenotypic effect.

Given the large number of targets for each miRNA, the deleterious effects can be due to a small number of strong effect genes or a large number of weak effect targets. Our recent paper addresses this issue[1]. Nevertheless, the underlying mechanistic wiring is not our main point. We simply suggest that, whatever the wiring is, its total effect is deleterious.

For possible mechanism of *mir-977*'s phenotypic effect. First, we observe *mir-977* KO shows advantage in reducing female receptivity; besides, *mir-977* KO shows advantage in increasing fertility by increasing egg production. There are two possible explanations that could be mutually reinforcing. A more plausible one is sperm themselves. In a separate submission (Lu et al. under review), we show that many *de novo* miRNAs affect sperm length. A second possibility is the seminal fluid proteins (Chapman et al. 2003; Ram and Wolfner 2005) as *mir-977* gene is weakly expressed in the accessory gland. Both the sperm and the seminal fluid are known to affect females' egg laying and receptivity.

References:

1. Zhao Y, Shen X, Tang T, Wu C-I: **Weak Regulation of Many Targets Is Cumulatively Powerful—An Evolutionary Perspective on microRNA Functionality**. *Mol Biol Evol* 2017, **34(12)**:3041-3046.
